# A Low-Cost Instrumentation System for Seismic Hazard Assessment in Urban Areas

**DOI:** 10.3390/s21113618

**Published:** 2021-05-22

**Authors:** Vassilis K. Papanikolaou, Christos Z. Karakostas, Nikolaos P. Theodoulidis

**Affiliations:** 1 School of Civil Engineering, Aristotle University of Thessaloniki, 54124 Thessaloniki, Greece; 2 Institute of Engineering Seismology and Earthquake Engineering (ITSAK-EPPO), 55102 Thessaloniki, Greece; christos@itsak.gr (C.Z.K.); ntheo@itsak.gr (N.P.T.)

**Keywords:** strong motion, MEMS, low-cost accelerograph, instrumentation, seismic hazard, shakemaps

## Abstract

The development and application of a low-cost instrumentation system for seismic hazard assessment in urban areas are described in the present study. The system comprises a number of autonomous triaxial accelerographs, designed and manufactured in house and together with dedicated software for device configuration, data collection and further postprocessing. The main objective is to produce a detailed view of strong motion variability in urban areas, for at least light intensity strong motion events. The overall cost of the developed devices is at least ten times lower than the respective commercial units, hence their deployment as an ultra-dense network over the area of interest can be significantly cost-effective. This approach is considered an efficient complement to traditional microzonation procedures, which are typically based on relatively few actual recordings and the application of theoretical methodologies to assess the strong motion distribution. The manufactured devices adopt micro-electro-mechanical (MEMS) digital sensor technology for recording acceleration, whereas the accompanying software suite provides various configuration options, quick browsing, analyzing and exporting of the recorded events, as well as GIS type functionality for seamlessly producing explicit seismic hazard maps of the considered area. The evaluation of system performance was based on shaking table and real field comparisons against high accuracy commercial accelerographs. The study concludes with a real application of the proposed system in the form of an ultra-dense network installed at the city of Lefkada, an earthquake prone urban area in Greece, and the following compilation of explicit shakemaps.

## 1. Introduction

**Figure 1:**
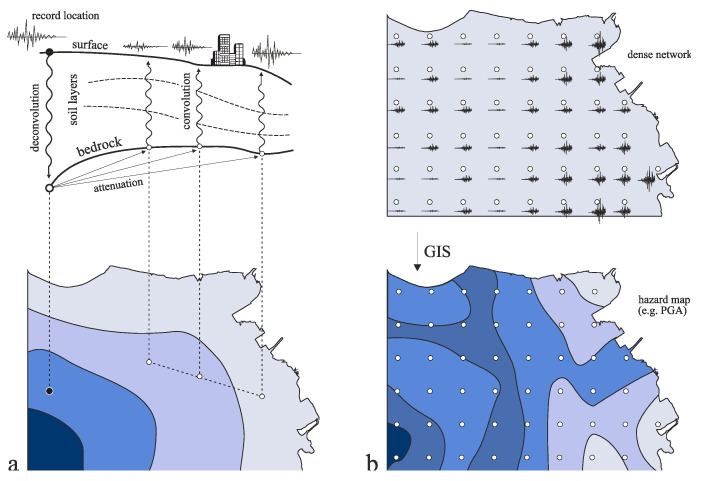
(**a**) Microzonation procedure and (**b**) suggested methodology.

It has long been recognized by the scientific community [1,2] that in case of a seismic event, a significant variation of the ground motion may be observed even within small distances due to various local geophysical and geological factors. However, even today, the existing national strong motion networks comprise a more or less limited number of high-accuracy accelerographs, mainly due to their high cost. The installation density of these commercial sensors within a country’s territory then has to be based on certain criteria (coverage of the total area, the variation of the seismic hazard and infrastructure exposure, maintenance capabilities, ease of access, etc.). These restrictions typically end up with the existence of one or only a few sensors even in the more important urban areas. The recordings of these networks are used, among others, to determine uniform seismic hazard zones within each nation’s territory with one representative seismic parameter for each zone. The latter, in the majority of modern seismic codes (e.g., [3]), is the “design peak ground acceleration” (PGA). However, for the design of important structures and infrastructure networks, as well as for seismic risk/loss studies of urban areas, the adoption of a single PGA value for the entire seismic hazard zone is inadequate, and recourse to microzonation studies is typically sought in order to assess the ground motion variability throughout the area of interest. Microzonation studies are based on actual recordings of strong motion within the region of interest (black dot, Figure 1a) and the knowledge of the underlying soil and bedrock profile, in order to evaluate the expected ground motion at other locations of interest (white dots, Figure 1a), through a deconvolution and eventual convolution of the actual recorded motion that involves, among others, the use of ground motion prediction equations. Despite its widespread application, the microzonation methodology has several inherent weaknesses: (i) as already mentioned before, only a limited number of acceleration recording stations are typically installed throughout the area of interest; (ii) the estimation of the site-specific response amplification from the actual recordings requires the application of numerical methods which require additional expensive experiments on the underlying bedrock and soil profiles (e.g., borehole/downhole measurements, microtremor measurements and several other geophysical and geotechnical investigations); and (iii) the overall reliability and accuracy of the results are not ensured, since they depend on the mapping detail of the underlying soil profiles throughout the area of study and the inherent uncertainties of the applied theoretical assumptions.

Especially for the case of urban areas, an ideal approach to explicitly obtain the desired seismic intensity distribution would be the deployment of an ultra-dense network of sensors throughout the area of interest (Figure 1b). The economic cost of such an implementation was, until recently, prohibitive, mainly due to the high cost of the high-accuracy commercial instruments, but also the significant respective installation, maintenance, and surveillance expenses. Consequently, the deployment of such dense accelerometer networks at a local scale is sparsely implemented worldwide (e.g., [4,5]). However, the deployment of even somewhat reduced accuracy sensors compared to the commercial ones would nevertheless provide the actual ground motion waveforms at each location, free of the uncertainties of the theoretical estimations of a microzonation study. This implementation would not only greatly improve the reliability of seismic risk estimation in the considered urban area (with all implied economic and social benefits), but would also contribute to further validation and optimization of existing analytical microzonation methods in future studies. Toward this aim, the design and manufacturing of autonomous accelerographs based on the newer low-cost micro-electro-mechanical sensor (MEMS) technology has rapidly grown in recent years (e.g., [6,7,8,9,10,11]), yet the manufacturing cost of these solutions varies significantly depending on the number of embedded features (e.g., sensor accuracy, processing power, connectivity, data streaming).

In the present study, since the main objective was to maintain a low cost for the production of the new device, an acceptable reduction in measurement accuracy compared to expensive commercial units was inevitable. Therefore, the research focused on optimizing cost-effectiveness, without compromising the basic requirements for earthquake engineering and engineering seismology applications, that is, capturing at least light intensity strong motion, with acceptable accuracy (e.g., PGA larger than approx. 0.014 g [12]). Furthermore, the development was also focused on simplicity and user friendliness in configuring and operating the low-cost device, as well as in data collection, assessment, and post-processing. It is also noted that in the suggested approach, the direct replacement of a defective device with a new one is economically feasible, preventing additional costs for repair facilities and personnel. In the following, a detailed presentation of the manufactured device and ad hoc software will be presented, along with the validation procedures using shaking table and real field comparisons against high accuracy commercial devices. The last section presents a real application of the proposed system in the form of an ultra-dense network installed at an earthquake prone urban area in the city of Lefkada, Greece, and the resulting compilation of explicit shakemaps. To the best of the authors’ knowledge, this was the first installation (in summer 2013) of an ultra-dense network of low-cost MEMS accelerographs at an urban area in Europe.

## 2. Hardware Development

**Figure 2:**
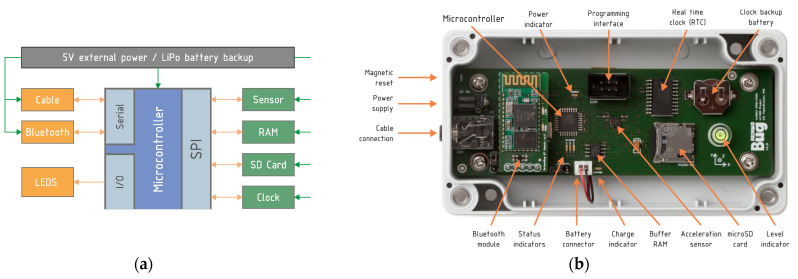
(**a**) Device architecture and (**b**) physical form.

The proposed seismic hazard assessment system designed and manufactured at the research unit of the Institute of Engineering Seismology and Earthquake Engineering, Greece (ITSAK) consists of a low cost accelerograph (SeismoBug^©^) and its dedicated software suite for configuration, data collection and general postprocessing. The device is an autonomous triaxial accelerograph, based on MEMS sensor technology, operating on external power supply with battery backup and featuring automatic event triggering capabilities, permanent local storage, pre-trigger buffer memory, accurate event time stamping, low-noise output, and compact size. The device circuitry is divided into three sections (Figure 2a), namely (i) the power section, based on a load sharing configuration, normally following an external 5V power source that switches automatically to battery power upon disconnection (e.g., due to a power outage during an earthquake); (i) the processing section, based on an 8-bit microcontroller running embedded firmware that interfaces with various peripherals; and (iii) the peripheral section, consisting of a 14-bit triaxial digital acceleration sensor [13], 1 Mbit random-access omemory (SRAM), non-volatile storage (2 GB microSD card) and an accurate real-time clock. The device in physical form is depicted in Figure 2b and its full specifications are presented in Table 1.

The device firmware was programmed in C and embedded in the microcontroller memory. Special attention was given to time-sensitive functions for interfacing with bus-shared peripherals to ensure minimum latencies, efficient strong-motion triggering algorithms (e.g., STA/LTA) and fast communication with host software.

## 3. Software Development and Device Validation

Following hardware design and manufacturing, a dedicated software suite comprising three different tools was developed for configuring various device parameters, retrieving stored data and postprocessing both at record-level (signal processing) as well as site-level (compilation of seismic hazard maps).

### 3.1. SeismoBug^©^ Monitor

**Figure 3:**
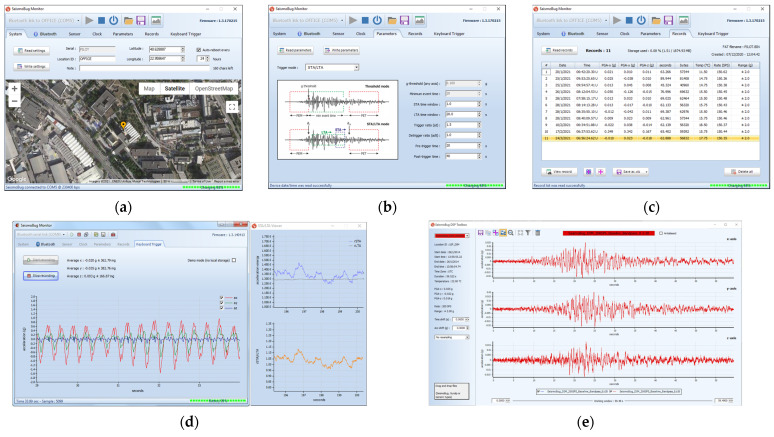
The SeismoBug^©^ Monitor software: (**a**) device settings (**b**) triggering settings (**c**) data retrieval (**d**) real-time mode (**e**) postprocessing.

The SeismoBug^©^ Monitor host software (Figure 3) supports device configuration, data retrieval, real-time monitoring and signal processing capabilities. Configurable parameters include device identification, georeferencing, connection security, sensor settings (e.g., sampling rate), real-time clock synchronization to NTP server, and strong motion triggering algorithms (absolute acceleration threshold or STA/LTA) with user-defined pre- and post-event recording periods. Moreover, the recorded data stored in local memory can be quickly transferred via serial cable or Bluetooth to a host PC and stored in readable spreadsheet format. The device can also be remotely operated by the host in keyboard-triggering mode for continuous acceleration monitoring. Finally, a few signal processing tools are also featured for quick viewing, trimming and comparing of multiple records both in time and frequency domains.

**Figure 4:**
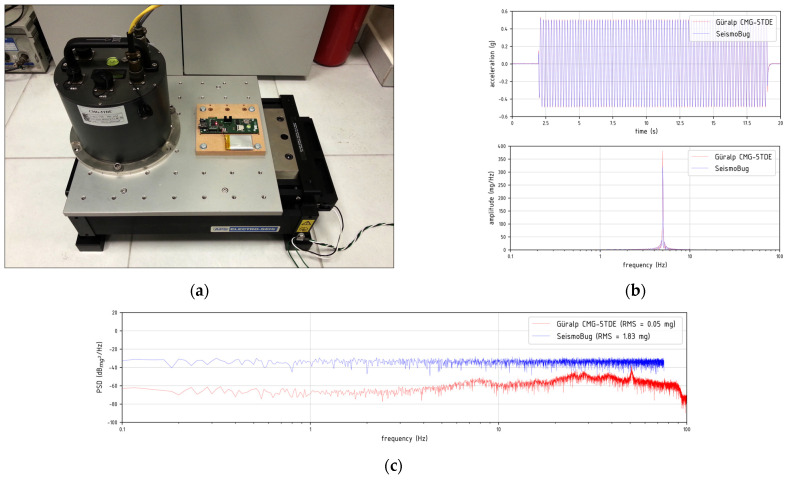
Device validation: (**a**) Seismic table test setup (**b**) Record comparison in time and frequency domain for a 5 Hzharmonic signal (**c**) PSD levels for a 60 s of ambient noise measurement.

Using the host software in keyboard triggering mode, the root mean square (RMS) of the output noise was measured lower than 2 mg for a standard 150 Hz output data sampling rate (ODR). An integrated 2nd order Butterworth digital lowpass filter at ODR/2 is pre-applied at the output. This is close to the inherent sensor noise specified by the manufacturer (see Table 1) and it implies a successful board design, since other undesirable noise sources (e.g., power supply, digital buses, grounding) could co-exist. The prototype units were then validated at the ITSAK laboratory to test the output accuracy both in terms of time and acceleration readings for various harmonic and random excitations at different frequency levels. For the testing, each device was mounted collinearly (along each of their axes) to a high resolution broad-band commercial accelerograph (Güralp^©^ Systems CMG-5TDE), on a uniaxial shaking table (Figure 4a). The correlation was excellent (see Figure 4b); however, for lower excitation levels, the higher noise level of the MEMS sensor in the form of power spectral density [14] is noticeable (Figure 4c). Nonetheless, in the subsequent sections it will be clearly demonstrated that, in real field applications, the device’s overall performance is more than adequate for capturing moderate to strong near field earthquake intensity measures. A more detailed presentation of the validation procedure is available in [15].

### 3.2. SeismoBug^©^ Browser

**Figure 5:**
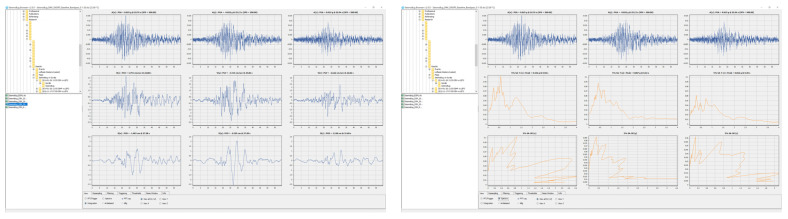
The SeismoBug^©^ Browser software.

The SeismoBug© Browser software was developed in the simple form of a “file explorer” (Figure 5) for the assessment and postprocessing of the bulk of records collected from the installed devices. The user can quickly browse through the record list and view various information for each one, helpful for quick identification and assessment. Specifically, the program displays the time–acceleration, velocity and displacement histories for all three directions, their decomposition in the frequency domain (FFT), elastic response spectra and triggering parameters (e.g., STA/LTA). Furthermore, direct record resampling (Lanczos) can be applied, together with linear baseline correction and bandpass filtering.

### 3.3. SeismoBug^©^ Manager

**Figure 6:**
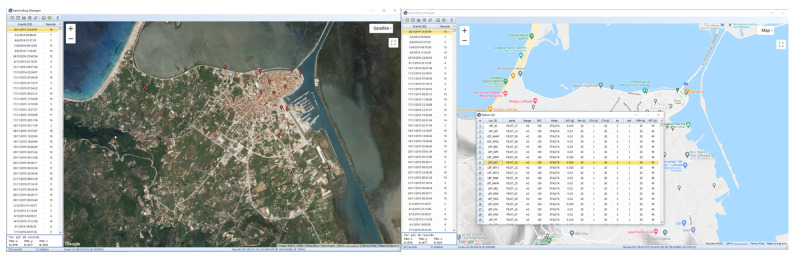
The SeismoBug^©^ Manager software.

For the compilation of ground motion shakemaps after a successful recording of an earthquake, the bulk of collected records from the installed network are postprocessed using the SeismoBug^©^ Manager GIS-based software (Figure 6). The installed unit locations are georeferenced on a digital map (Google Maps^©^) and all concurrent records (within a reasonable time window) are automatically grouped as “events”. The software links to the EMSC database [16], allowing the user first to verify whether a displayed “event” corresponds to an actual earthquake and then retrieve the relevant characteristics (epicenter, magnitude, depth, etc.). Using a seamless integration with a third-party contour mapping software [17], the event data can be displayed in various forms of intensity distribution maps (see in Section 4). The parameter selected for compiling the above intensity maps is the peak ground acceleration (PGA), since it is the one typically adopted seismic intensity measure in current seismic codes. However, within the same software framework, more elaborate ground motion parameters (e.g., Arias intensity) can be easily implemented.

## 4. System Application

In this section, a recent application of the low-cost instrumentation system is described in order to assess its overall performance under real field conditions. For this application, 21 low-cost SeismoBug^©^ stations were installed in the greater urban area of the city of Lefkada (highest seismicity zone; PGA = 0.36g [1]), located in the northeastern part of the Lefkada island.

### 4.1. Installation

**Figure 7:**
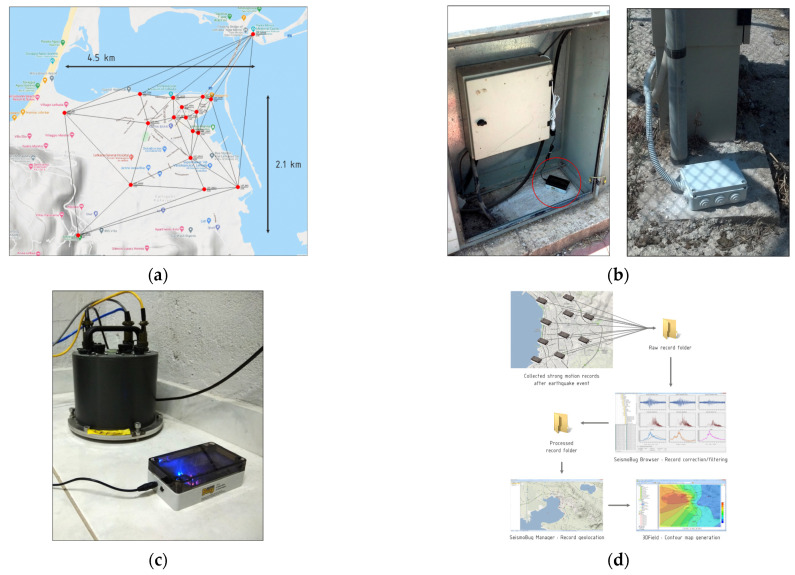
The deployed ultra-dense network (**a**) installation locations (**b**) free-field installation (**c**) basement installation (**d**) outline of the data collection, postprocessing and mapping procedure.

The network was deployed within an untypically small area, forming an ultra-dense grid (red dots) with a maximum distance between installation locations of approximately 1200 m and a minimum distance of less than 150 m (Figure 7a). The majority of the units were installed inside electricity feeder pillars (metallic enclosures that provide electric power to streetlights) or in waterproof electrical junction boxes on the pillar concrete base (Figure 7b), in order to represent free-field conditions. About one third of the units were installed inside buildings, mainly due to the unavailability of suitable free-field conditions at the desired location. For comparison purposes, two units were intentionally placed next to high-accuracy accelerographs (Güralp^©^ Systems CMG-5TDE, identical to the one used for initial validation), one of the National Strong Motion Network operated by ITSAK, at the basement of the Prefecture building of Lefkada (Figure 7c), and an additional one installed within the framework of the present research effort in free-field, stand-alone conditions, at a distance of approximately 80 m from the building. Figure 7d depicts a typical outline of the data collection and postprocessing after the occurrence of a seismic event, using the software suite presented in the previous sections. A more detailed presentation on the network deployment is available in [18].

The installed devices were synchronized to a common time reference, yet within the limitation set by their real-time clock accuracy (2 ppm, see Table 1). Small time drifts were observed during regular maintenance visits; however, they did not affect the identification of “common” seismic events. Moreover, all units were initially configured to use the absolute acceleration threshold triggering scheme; however, this was found to be prone to false triggering and they were soon switched to a standard STA/LTA algorithm, implemented in a device firmware update.

### 4.2. Network Operation

**Figure 8:**
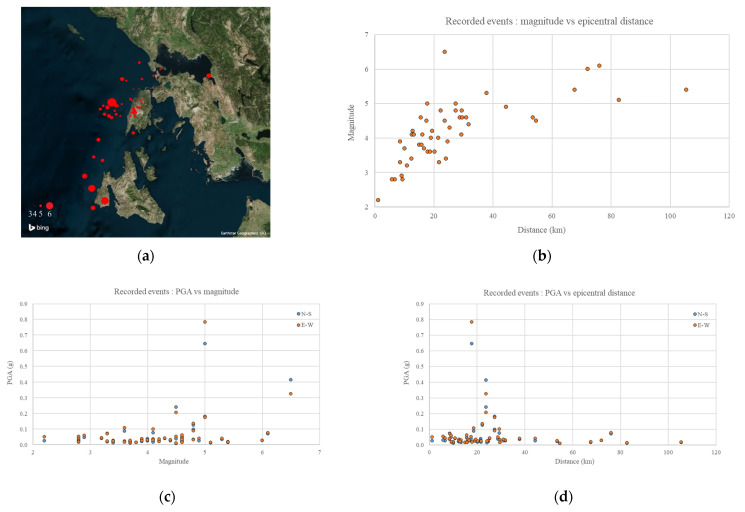
Recorded earthquakes (**a**) epicenters, (**b**) magnitude vs. distance, (**c**) PGA vs. magnitude, (**d**) PGA vs. distance.

Since its initial installation in 2013, the Lefkada network has been running for thousands of operating hours with zero device failures and was triggered by more than 50 light to strong intensity measure earthquakes in the broader area (red dots in Figure 8, size relative to the respective magnitude; network area is displayed by a yellow circle). In Figure 8b, the magnitude vs. epicentral distance of the recorded earthquakes is depicted, indicating the detectability of the Lefkada network. Figure 8c,d show the recorded PGAs (N–S and E–W directions) against magnitude and epicentral distance, respectively, indicating the network sensitivity.

**Figure 9:**
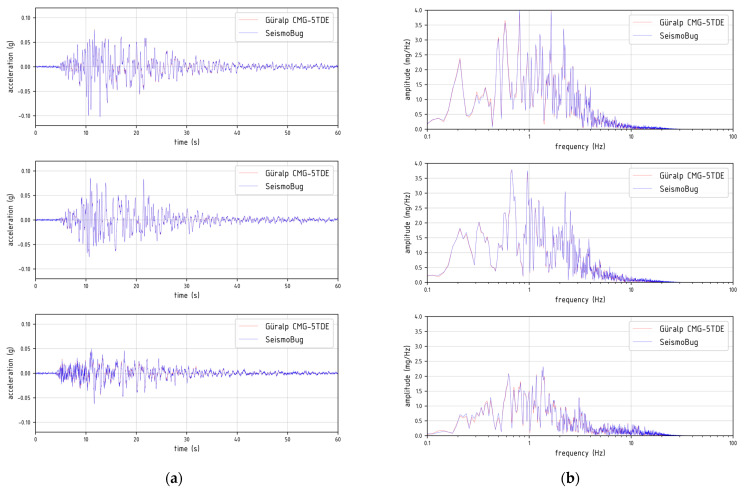
Comparison of acceleration records for the M6.5 Lefkada earthquake between SeismoBug^©^ and Güralp^©^ Systems accelerographs in (**a**) time and (**b**) frequency domains (components X, Y, Z from top to bottom).

The excellent performance of the low-cost devices was confirmed by acceleration record comparisons between the aforementioned adjacent SeismoBug^©^ and Güralp^©^ units installed at the Lefkada Prefecture building. Figure 9 depicts one of these comparisons at the building basement (see Figure 7c) after the strong M6.5 Lefkada earthquake (17 November 2015 07:10:08 UTC). It is observed that accelerations (and hence PGA) recorded by the two devices are practically identical, for earthquake engineering purposes, both in time and frequency domains. Only in very low levels of acceleration, the inherent noise of the low-cost sensor is noticeable. Despite this fact, as well as the lack of expanded capabilities incorporated in the commercial unit (such as GPS time and real-time telemetric data transmission), it is noted that the possible commercial cost of the SeismoBug© device would be at least an order of magnitude less than that of Güralp© or a similar high-resolution unit.

**Figure 10:**
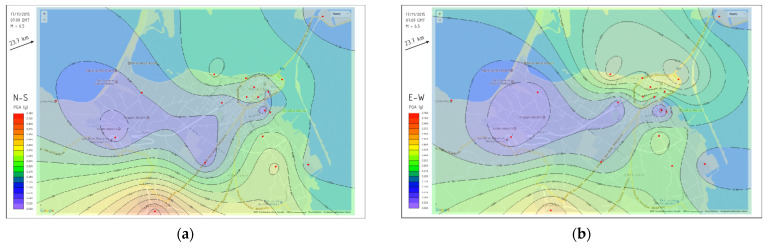
PGA contour maps (**a**) N-S and (**b**) E-W directions for the M6.5 Lefkada earthquake.

As already described earlier, the collected acceleration data can be, among others, properly postprocessed with the custom-developed software suite to produce local PGA distribution maps for each recorded earthquake event. The respective shakemaps for the M6.5 Lefkada earthquake are presented in Figure 10 for both horizontal directions (N–S and E–W). This functionality clearly demonstrates the added value of employing efficient low-cost devices in dense urban networks as a complementary effort towards seismic risk mitigation and ground shaking consequences on the built environment. A more detailed presentation of other recorded earthquakes, compiled shakemaps and their implications from an earthquake engineering point of view is available in [19].

The advantage of the proposed methodology becomes evident in Figure 10: the only existing high-accuracy (Güralp©) accelerograph of the National Strong Motion Network (at the building basement) recorded a PGA (E–W) value of 0.084 g (N–S direction), and this value would be typically used as the representative one for the whole area, while the actual PGA values recorded by the low-cost dense network throughout the area ranged from 0.074 g to 0.326 g (E–W direction). Such concrete quantitative information would be very difficult and expensive to obtain through the application of conventional methods, such as a microzonation study, and even then the accuracy of the estimated results would still have a margin of uncertainty, depending on the specific assumptions that would have to be inevitably used.

## 5. Conclusions and Further Developments

In this study, a low-cost instrumentation project has been presented, based on low-cost accelerographs designed and manufactured in-house, supported by a dedicated software suite. The main objective was to capture a detailed view of strong motion distribution in urban areas during seismic events of light to strong intensity. The developed devices were used to implement an ultra-dense network of 21 stations in a high-seismicity urban area in Greece, which has already recorded a large number of strong motion data. The presented results demonstrate that the developed low-cost MEMS device makes it economically feasible to set up ultra-dense grid networks in areas or infrastructure of interest (e.g., urban ones, lifelines in rural areas) in order to obtain the actual variation of the seismic excitation during various earthquake events. It can also significantly contribute to scientific studies of various subjects in the field of Engineering Seismology and Earthquake Engineering. Another advantage of the low-cost units is that the direct replacement of a defective device with a new one is practical, preventing additional costs for repair facilities and personnel. The proposed approach is an effective and promising complementary effort towards seismic risk mitigation and consequences of earthquake ground motion on built environment and critical infrastructures.

**Figure 11:**
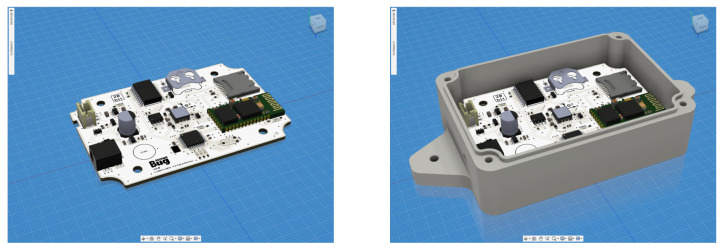
Second generation of the SeismoBug^©^ device under development (photorealistic model).

The herein presented instrumentation proposal is part of an ongoing effort to further expand and refine the capabilities of the low-cost device, not only by taking advantage of the ever-evolving technology but also by maintaining a balance between performance and production cost. In this respect, a second generation of the SeismoBug^©^ device is currently under development, featuring enhanced sensor characteristics, new wireless transfer protocols, real-time data continuous data transmission and 40% smaller form factor (Figure 11).

## 6. Patents

The herein described device has obtained a patent from the Hellenic Industrial Property Organization (OBI) (“Autonomous triaxial accelerograph”, patent no. 1008498).

## Figures and Tables

**Table 1:** Device specifications.

Feature	Description
Acceleration sensor	Bosch Sensortec BMA180 14-bit Nominal noise 150 μg/√Hz, measured ≈ 18 mg RMS @ 150 SPS 2nd order Butterworth digital filter at half output data rate
Microcontroller	Atmel ATmega328P 3.3V@11.0592 MHz
Buffer memory	1 Mbit SRAM
Real-time clock	Battery operated RTC with 2 ppm accuracy (max 0.17 s/day, ≈1 min/year)
Local storage	microSD card 2 GB
Connectivity	Serial cable or wireless Bluetooth @230.4 Kbps
Power	Power supply 5 V/1 A and LiPo backup battery 3.7 V 850 mAh
Power consumption	External power: 11.5/53.5/18.5 mA (cable only/BT standby/BT paired) Battery: 7.35/49.5/14.5 mA (cable only/BT standby/BT paired)
Dimensions	120 × 65 × 40 mm
Weight	170 g

## Data Availability

Not applicable.
